# Parental Competencies as Familial Protective Factors in Adolescent Substance Use Prevention: A Scoping Review

**DOI:** 10.3390/children13091193

**Published:** 2026-09-04

**Authors:** Kyung-Young Hong, Jihye Shin

**Affiliations:** 1 College of Nursing, Eulji University, Uijeongbu-si 11759, Gyeonggi-do, Republic of Korea; hong2813@eulji.ac.kr; 2 College of Nursing, Catholic University of Pusan, Busan 46252, Republic of Korea

**Keywords:** adolescent, substance use prevention, parental competency, parenting, family, protective factors, scoping review

## Abstract

**Highlights:**

**What are the main findings?**

- Thirty-two publications identified 11 parental competency domains relevant to adolescent substance use prevention.
- Relational bonding, prevention communication, monitoring, and rule-setting were the most frequently represented competencies.
- Parental competencies were more often targeted in interventions than directly measured or examined in relation to adolescent substance-use outcomes.

**What are the implications of the main findings?**

- Prevention programs should explicitly define and strengthen modifiable parental competencies rather than broadly targeting parenting or family functioning.
- Standardized measures are needed to assess distinct parental competencies and their changes following prevention interventions.
- Future research should examine whether specific parental competencies contribute to adolescent substance-use outcomes and function as protective mechanisms.

**Abstract:**

Parents play an important role in adolescent substance use prevention, yet the competencies underlying their protective role remain inconsistently conceptualized and measured. This scoping review aimed to map parental competencies as modifiable family-level protective factors for adolescent substance use prevention. Following Joanna Briggs Institute methodology and PRISMA-ScR guidance, nine electronic databases were searched from inception to 20 July 2026. Peer-reviewed original studies addressing parental competencies relevant to substance use prevention among adolescents aged 9–19 years were included. Competencies were mapped according to their conceptual domains and evidence roles. Thirty-two publications were included, identifying 11 parental competency domains. Relational bonding and family involvement (90.6%), prevention communication (87.5%), monitoring and supervision (62.5%), and rules, boundaries, and expectations (53.1%) were most frequently represented. Competencies were more often targeted in interventions than directly measured or examined in relation to adolescent substance-use outcomes, and measurement approaches varied considerably across studies. Parental competencies relevant to adolescent substance use prevention encompass multiple relational, communicative, supervisory, and behavioral capacities. The findings highlight important gaps between intervention content, competency measurement, and outcome-related evidence and provide a foundation for developing competency-based prevention interventions and measurement tools.

## 1. Introduction

Adolescence is a critical developmental period during which increasing autonomy, peer influence, and opportunities for experimentation may contribute to the initiation and escalation of substance use. Early substance use is associated with an increased likelihood of later substance-related problems and may occur alongside academic, behavioral, psychological, and social difficulties [1]. Prevention during this period therefore requires attention not only to adolescents’ individual knowledge and refusal skills but also to the family and social environments in which substance-related attitudes and behaviors develop.

Adolescent substance use is influenced by interacting individual, familial, social, cultural, and environmental conditions. Recent family-based research demonstrates that parents’ characteristics may contribute to adolescents’ vulnerability; for example, parental psychopathology has been associated with an increased likelihood of substance use disorder among children and adolescents [1]. At the same time, structural disadvantage, neighborhood violence, and low collective efficacy may shape the environments in which parents attempt to protect their children. Differences between parent and adolescent perceptions of neighborhood risks also suggest that effective prevention may require communication about risks extending beyond the immediate household [2].

Parents and primary caregivers can nevertheless serve as important protective resources. Through warmth, involvement, communication, monitoring, behavioral expectations, and role modeling, parents may reduce adolescents’ exposure to risk and support the development of adaptive coping and decision-making. Longitudinal evidence suggests that early parental involvement and expectations may influence later substance-related outcomes through developmental pathways involving adolescents’ competencies and behavioral adjustment [3]. Accordingly, parents may contribute to prevention both through their immediate actions and through longer-term effects on adolescent development.

Parent- and family-focused prevention programs have attempted to strengthen these protective processes. A systematic review of parenting programs found that interventions emphasizing active parental involvement and the development of parenting and self-regulation skills showed promise for preventing or reducing tobacco, alcohol, and drug use among young people [4]. More recent interventions have incorporated culturally tailored parent education, family communication, and family functioning into substance use prevention for Latinx adolescents [5]. These developments indicate a continuing shift from providing parents with information alone toward strengthening the skills and family processes required to support adolescent health.

Parent-focused prevention may be particularly important for adolescents experiencing adversity. Justice-involved youth frequently encounter trauma, disrupted relationships, and elevated opioid or other substance-use risks. Contemporary caregiver-based approaches therefore emphasize trust, emotional and instrumental support, role modeling, communication, behavioral boundaries, and trauma-informed responses [6]. These approaches illustrate that preventive parenting may require context-specific judgment and relational skills in addition to general supervision.

Cultural context may also determine how parental protection is understood and practiced. A culturally grounded program for Navajo girls and their female caregivers incorporated intergenerational participation, cultural connectedness, parent–child communication, and family engagement [7]. Although the participating children were younger than the population of the present review, the study demonstrates that culture and collective family practices may constitute active protective resources rather than merely contextual characteristics. Similar principles have informed Indigenous family-based substance use prevention programs involving kinship systems, Elders, cultural identity, and community participation [8,9].

Despite the recognized importance of parents, the literature does not consistently define the capacities parents require to prevent adolescent substance use. Existing studies have examined related constructs such as parenting competence, parenting self-efficacy, family functioning, parental monitoring, communication, discipline, support, and relationship quality. However, these constructs differ in scope and frequently represent either general parenting abilities or isolated parenting behaviors. They do not necessarily capture the integrated knowledge, skills, judgment, self-efficacy, and actions required to recognize substance-related risks, communicate preventive expectations, respond to warning signs, and obtain appropriate support.

The measurement of these parental capacities has also remained fragmented. Some studies have separately assessed communication, monitoring, discipline, role modeling, and anti-drug messages [10]. Others have measured prevention self-efficacy or parents’ perceived influence over their child’s substance use [11,12]. Broader measures of family relationships or positive parenting have combined multiple parental behaviors into composite constructs [13,14]. Although these approaches provide important evidence, the resulting conceptual and measurement heterogeneity makes it difficult to determine which parental competencies have been identified, how they have been operationalized, and which have been examined as protective factors for adolescent substance use.

The distinction between a parenting characteristic and a parental competency is important. Parental psychopathology, parental substance use, socioeconomic adversity, and neighborhood disadvantage may affect adolescent substance-use risk, but they are not necessarily competencies that parents can acquire or develop. Conversely, knowledge of substance-related risks, effective communication, monitoring, rule setting, prevention planning, and resource seeking may represent modifiable capacities. Relational warmth and culturally responsive parenting may also be considered competencies when they are operationalized as developable and observable parental practices rather than as fixed family characteristics.

Existing reviews have primarily evaluated whether parenting or family programs reduce adolescent substance use [4]. Such evaluations are essential, but program effectiveness alone does not clarify which parental competencies were targeted, whether parents acquired them, or whether changes in those competencies were associated with adolescent outcomes. Multicomponent interventions may simultaneously address communication, monitoring, discipline, family bonding, adolescent refusal skills, and broader contextual factors. Consequently, a favorable intervention outcome cannot be attributed to a particular parental competency without direct measurement or mechanism analysis.

A scoping review is appropriate for this field because the relevant evidence includes intervention trials, observational studies, program-development research, cultural-adaptation studies, feasibility evaluations, and implementation analyses. These heterogeneous sources can be used to map how parental competencies have been conceptualized and operationalized without restricting the synthesis to a single study design or outcome. A scoping approach also permits examination of the gap between competencies included as intervention content, competencies directly measured in parents, and competencies statistically examined in relation to adolescent substance-use outcomes.

Therefore, this scoping review aimed to map parental competencies identified, measured, or targeted as modifiable family-level protective factors for preventing substance use among adolescents aged 9–19 years. Specifically, the review addressed the following questions:

1. What parental competency domains have been identified, measured, or targeted in the literature on adolescent substance use prevention?
2. How have these competencies been operationalized and measured across different study designs, populations, and intervention contexts?
3. To what extent have the identified competencies been included as intervention targets, directly measured as parental capacities, or statistically examined in relation to adolescent substance-use outcomes?
4. What conceptual, measurement, and empirical gaps should be addressed in future parent- and family-focused prevention research?

In this review, substance use encompassed alcohol, tobacco and nicotine products, cannabis, prescription drug misuse, inhalants, and other illicit substances. Parental competency was understood as an integrated and potentially developable capacity involving knowledge, skills, judgment, self-efficacy, and behavior used by parents or primary caregivers to prevent adolescent substance use within family and parent-involved contexts.

## 2. Methods

### 2.1. Protocol and Reporting Guideline

The protocol for this scoping review was prospectively registered with the Open Science Framework (OSF; https://osf.io/njxwu/ accessed on 31 August 2026). The review was conducted in accordance with the Joanna Briggs Institute (JBI) methodology for scoping reviews and is reported following the Preferred Reporting Items for Systematic Reviews and Meta-Analyses extension for Scoping Reviews (PRISMA-ScR). The completed PRISMA-ScR checklist is provided in Supplementary Table S1.

### 2.2. Eligibility Criteria

Eligibility criteria were developed using the Population–Concept–Context (PCC) framework recommended by the Joanna Briggs Institute (Table 1). Studies were eligible if they examined parental competencies relevant to the prevention of substance use among adolescents aged 9–19 years. Although adolescence is commonly defined as ages 10–19 years, the lower age limit was extended to 9 years to accommodate preventive studies with mixed-age samples that included children transitioning into early adolescence. Such studies were retained when the sample predominantly or substantively represented the target adolescent period or provided age-relevant evidence on parental competencies for substance use prevention. This broader inclusion also reflects the preventive focus of the review, as parent- and family-focused interventions may begin before substance-use behaviors become established. Parents and primary caregivers were the primary population of interest; however, studies in which adolescents reported or evaluated relevant parental competencies or parenting practices were also eligible.

The concept of interest was parental competency, defined for this review as parental knowledge, skills, judgment, self-efficacy, and behaviors that were identifiable, measurable, or modifiable as family-level protective factors for adolescent substance use prevention. Studies were eligible when such competencies were directly measured, examined in relation to adolescent substance-use outcomes, or explicitly targeted within a preventive intervention. Constructs representing relatively nonmodifiable parental or contextual characteristics, such as socioeconomic status, family structure, parental psychopathology, or neighborhood disadvantage, were not classified as parental competencies. Parental substance use was also not considered a competency in itself; however, modifiable parenting practices related to substance use, such as parental role modeling, communication of substance-related expectations, or establishment of family rules, were eligible.

Eligible contexts included family and home settings and parent-involved school, community, clinical, and digital prevention settings, with no geographical restrictions. Substance use encompassed alcohol, tobacco and nicotine products, cannabis, prescription drug misuse, inhalants, and other illicit substances. Studies addressing universal, selective, or indicated prevention were considered, whereas studies focused exclusively on the treatment of established substance use disorders without a relevant preventive parental component were excluded.

A broad range of empirical evidence was eligible, including observational and intervention studies using quantitative, qualitative, or mixed-methods designs. Peer-reviewed original research articles published in English were included. Reviews, meta-analyses, study protocols without empirical findings, editorials, commentaries, conference abstracts, dissertations, and other non-peer-reviewed publications were excluded.

### 2.3. Search Strategy

A comprehensive literature search was conducted across nine electronic databases: PubMed/MEDLINE, Embase, CINAHL, PsycINFO, Web of Science, KMbase, DBpia, KISS, and RISS. The databases were searched from inception to 20 July 2026, with no restrictions on publication year. The search strategy was designed to identify literature addressing parental competencies and modifiable parenting practices relevant to adolescent substance use prevention.

Search terms were developed around five core concepts: (1) adolescents; (2) parents, caregivers, and parenting; (3) parental competencies and modifiable parenting practices, such as communication, monitoring, supervision, rule-setting, support, and self-efficacy; (4) substance use, including alcohol, tobacco and nicotine products, cannabis, prescription drug misuse, and other substances; and (5) prevention. Controlled vocabulary, including Medical Subject Headings (MeSH) where applicable, was combined with free-text terms. Search terms and syntax were adapted to the indexing systems and requirements of each database, and Boolean operators (AND and OR) were used to combine concepts and related terms.

**Figure 1:**
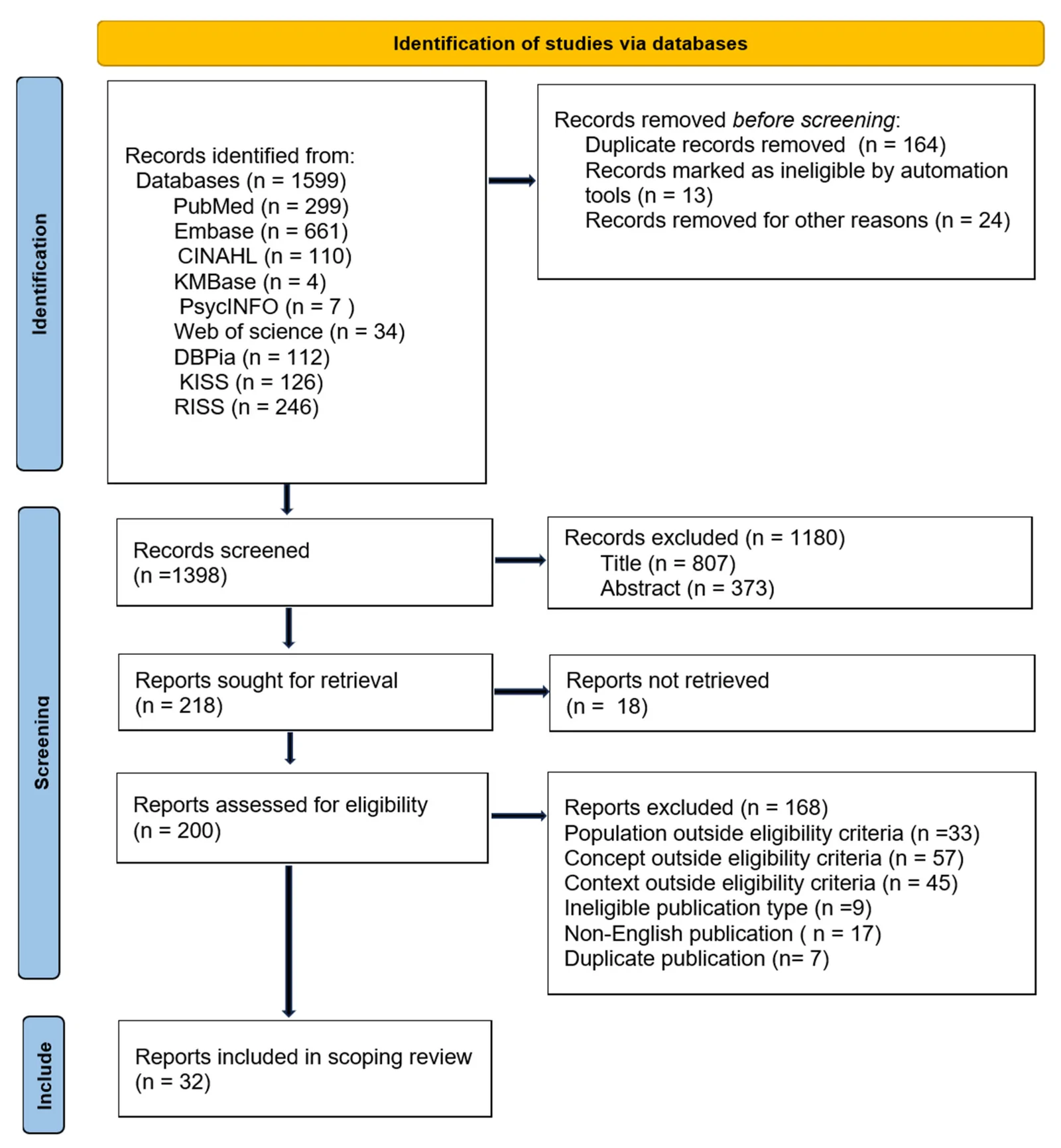
PRISMA flow of study selection process.

No geographical restrictions were applied. Only peer-reviewed original research articles published in English were considered eligible for inclusion. The complete search strategies are provided in Supplementary Table S2. The study identification and selection process is presented in Figure 1.

### 2.4. Study Selection

All records identified through the database searches were imported into a reference management program, and duplicate records were removed before screening. Study selection was conducted in two stages according to the predefined eligibility criteria. Two reviewers independently screened the titles and abstracts of the retrieved records to identify potentially relevant publications. The full texts of potentially eligible reports were subsequently retrieved and independently assessed by the same two reviewers against the Population–Concept–Context (PCC) criteria described above. Disagreements at either stage were resolved through discussion and consensus.

At the full-text stage, reasons for exclusion were recorded and categorized according to the predefined eligibility criteria, including population outside the eligibility criteria, concept outside the eligibility criteria, context outside the eligibility criteria, ineligible publication type, non-English publication, and duplicate publication.

The study selection process is presented in the PRISMA flow diagram (Figure 1). Of the 1599 records identified through database searching, 200 reports underwent full-text eligibility assessment, and 32 reports were ultimately included in the scoping review.

### 2.5. Data Charting and Extraction

A standardized data-charting form was developed based on the review questions and was used to systematically extract relevant information from the included reports. The charted data included publication characteristics (author, year, and country), study design and setting, participant characteristics, adolescent age range, sample size, substance-use focus, and characteristics of parent- or family-involved interventions where applicable. Information regarding the parental competencies identified, measured, or targeted in each report was also extracted, together with the measures or indicators used to operationalize these competencies and their reported relationships with adolescent substance-use outcomes, when available.

Two reviewers independently charted the data from the included reports. The extracted information was compared, and discrepancies were resolved through discussion and consensus. The data-charting form was refined iteratively during the review process when additional relevant categories emerged from the included literature, while maintaining consistency with the predefined review questions and operational definition of parental competency.

For competency mapping, parental constructs and practices reported in the included studies were extracted using the terminology of the original publications and subsequently grouped into conceptually related competency domains. Constructs were classified as parental competencies when they represented potentially developable parental knowledge, skills, judgment, self-efficacy, or behaviors relevant to adolescent substance use prevention. Nonmodifiable parental or contextual characteristics were not coded as competency domains. When a report addressed more than one parental competency, each relevant competency was charted separately.

### 2.6. Data Synthesis and Competency Mapping

Given the heterogeneity of the included evidence sources in study design, intervention characteristics, parental constructs, measurement approaches, and substance-use outcomes, the extracted data were synthesized descriptively rather than quantitatively. Publication characteristics were summarized using frequencies and percentages, together with a narrative description of study designs, populations, intervention characteristics, and follow-up periods. The publication was used as the primary unit of descriptive mapping. When multiple publications originated from the same underlying trial or prevention program, they were retained as separate evidence sources because they contributed distinct information regarding intervention content, measurement, mechanisms, or follow-up; however, their shared study origin was considered when interpreting the evidence.

Parental constructs identified in the included publications were mapped into conceptually related competency domains through an iterative process of comparison and grouping. Constructs were classified according to their underlying preventive function rather than solely according to the terminology used in the original publications. A construct was mapped as a parental competency when it represented potentially developable parental knowledge, skills, judgment, self-efficacy, or behavior relevant to adolescent substance use prevention. General family characteristics, parental risk factors, contextual or implementation factors, intervention outcomes, and adolescent competencies were not classified as parental competency domains. Each publication could contribute to more than one domain, and each competency domain was counted once per publication. Accordingly, competency domains were not mutually exclusive, and percentages were calculated using the 32 included publications as the denominator.

To distinguish the role of each competency within the evidence base, competency domains were further classified into three non-mutually exclusive evidence categories: targeted, when the competency was explicitly incorporated as intervention content; measured, when it was directly assessed as a parent or caregiver construct; and associated, when it was statistically examined in relation to an adolescent substance-use outcome. Classification as associated indicated that a statistical relationship was examined and did not necessarily indicate statistical significance, causality, or an intervention effect.

Measurement approaches were additionally synthesized according to the constructs assessed, informants, degree of substance specificity, and use of individual versus composite parenting measures. Associations between parental competencies and adolescent substance-use outcomes were synthesized narratively because of substantial variation in study design, developmental timing, competency operationalization, and substance-use outcomes. No meta-analysis or other inferential pooling was undertaken.

## 3. Results

### 3.1. Characteristics of the Included Publications

Following the screening and eligibility assessment, 32 publications met the predefined eligibility criteria and were included in the scoping review (Figure 1). The included publications represented diverse study designs, populations, and parent- or family-involved approaches to adolescent substance use prevention (Supplementary Table S3).

The 32 publications represented at least 28 distinguishable study or program clusters. Several publications originated from the same underlying trials but addressed different intervention components, follow-up periods, mediating mechanisms, or implementation processes. Specifically, publications [13,15,16] originated from the same web-based mother–daughter prevention trial; publications [17,18] were derived from the same Parents Who Care/Staying Connected with Your Teen trial; and publications [19,20] originated from the original Strong African American Families (SAAF) efficacy trial. Publications [8,9] addressed related stages in the cultural adaptation of the Strengthening Families Program for Indigenous families but were not treated as duplicate reports of a single outcome study. SAAF–T [21] was considered distinct from the original SAAF trial [19,20]. Accordingly, publications were retained as separate evidence sources for descriptive mapping, while shared study origins were considered when interpreting the findings. This approach was consistent with the publication-level unit of analysis used for competency mapping.

The evidence base included randomized controlled trials and other intervention studies, longitudinal and cross-sectional observational studies, and program-development, cultural-adaptation, feasibility, and implementation research. Intervention studies ranged from relatively brief parent-focused educational or communication interventions to multicomponent family-based prevention programs involving adolescents and their parents or caregivers. Delivery formats included face-to-face family sessions, parent groups, home-based activities, web-based programs, and other technology-supported approaches.

The included publications addressed adolescents across different developmental stages and involved diverse family, racial, ethnic, and cultural populations. Most studies were conducted in the United States, although evidence from other countries and culturally specific or Indigenous family contexts was also represented. Several studies focused on populations experiencing elevated substance-use or contextual risks, whereas others evaluated universal family-based prevention approaches.

The substance-use outcomes and prevention targets were similarly heterogeneous. Publications addressed alcohol, tobacco and cigarette use, electronic cigarettes or vaping, cannabis or marijuana, prescription drug misuse, and other illicit or unspecified substances, with several studies addressing multiple substances. Follow-up periods varied considerably across intervention studies, ranging from immediate post-intervention assessment to longitudinal follow-up extending over several years. Detailed characteristics of the included publications are presented in Supplementary Table S3.

### 3.2. Parental Competency Domains

Eleven parental competency domains were identified across the 32 included publications (Table 2). Because individual publications frequently addressed multiple aspects of parenting, each publication could be assigned to more than one competency domain. Accordingly, the domains were not mutually exclusive, and the percentages do not sum to 100%.

Relational bonding and family involvement (PC9) was the most frequently identified domain, appearing in 29 publications (90.6%), followed by prevention communication (PC2) in 28 (87.5%), monitoring and supervision (PC3) in 20 (62.5%), and rules, boundaries, and expectations (PC4) in 17 (53.1%). Together, these findings indicate that the literature predominantly addressed relational and behavioral parenting competencies involving supportive parent–adolescent relationships, communication, supervision, and the establishment of clear behavioral expectations.

Consistent discipline and behavior management (PC5) was identified in 13 publications (40.6%), while parental role modeling and family norms (PC7) appeared in eight (25.0%). Culturally responsive protective parenting (PC11) was identified in seven publications (21.9%) and encompassed developable parenting practices such as racial or cultural socialization, reinforcement of cultural identity, culturally grounded communication, and the mobilization of kinship or family resources. Cultural adaptation of an intervention alone was not sufficient for classification within this domain unless an observable or developable parental practice was identified.

Competencies more specifically related to parents’ prevention knowledge, confidence, planning, and navigation of resources were less frequently represented. Substance-related knowledge and risk awareness (PC1) was identified in six publications (18.8%), parental prevention self-efficacy (PC6) in four (12.5%), information and resource seeking (PC10) in four (12.5%), and prevention planning and commitment (PC8) in three (9.4%).

Overall, the mapping showed that relational bonding, communication, monitoring, and rule setting were the most commonly represented parental competencies, whereas prevention-specific self-efficacy, resource seeking, and explicit prevention planning were comparatively uncommon. These frequencies reflect the extent to which each competency was represented in the included literature and should not be interpreted as evidence of its relative importance or effectiveness. Detailed operational definitions, representative indicators, and publication-level frequencies for all 11 domains are presented in Table 2.

### 3.3. Evidence Roles and Measurement Gaps

The included publications differed in how parental competencies were represented within the evidence base (Table 3). Detailed publication-level evidence mapping across the 11 parental competency domains is provided in Supplementary Table S4. Competencies were classified as targeted when explicitly incorporated as intervention content, measured when directly assessed as a parent or caregiver construct, and associated when statistically examined in relation to an adolescent substance-use outcome. These evidence roles were not mutually exclusive. The associated classification indicates that a statistical relationship was examined and does not necessarily indicate statistical significance, causality, or intervention effectiveness.

The most extensively represented domains showed a consistent gap between intervention targeting and empirical assessment. Prevention communication (PC2) was the most frequently targeted competency, appearing as intervention content in 26 publications (81.3%), but was directly measured in 14 (43.8%) and examined in relation to adolescent substance-use outcomes in four (12.5%). Relational bonding and family involvement (PC9) was targeted in 24 publications (75.0%), measured in 18 (56.3%), and examined in relation to substance-use outcomes in seven (21.9%). Monitoring and supervision (PC3) was targeted in 18 publications (56.3%), measured in 12 (37.5%), and examined in relation to substance-use outcomes in three (9.4%).

A similar target-to-measurement gap was observed for behavioral management competencies. Rules, boundaries, and expectations (PC4) was targeted in 16 publications (50.0%) but measured in six (18.8%) and examined in relation to adolescent substance use in one (3.1%). Consistent discipline and behavior management (PC5) was targeted in 13 publications (40.6%), measured in six (18.8%), and examined in relation to substance-use outcomes in one (3.1%). Parental role modeling and family norms (PC7) was targeted in seven publications (21.9%), measured in two (6.3%), and examined in relation to adolescent substance use in one (3.1%).

Evidence was more limited for prevention-specific cognitive, planning, and resource-navigation competencies. Substance-related knowledge and risk awareness (PC1) was targeted in six publications (18.8%) but directly measured in only one (3.1%), with no publication examining its relationship with an adolescent substance-use outcome. Parental prevention self-efficacy (PC6) was targeted and measured in three publications (9.4%) but was not examined in relation to adolescent substance use. Prevention planning and commitment (PC8) was targeted in three publications (9.4%) and measured in one (3.1%), while information and resource seeking (PC10) was targeted in four publications (12.5%) but was not directly measured. Neither PC8 nor PC10 was statistically examined in relation to adolescent substance-use outcomes.

Culturally responsive protective parenting (PC11) was targeted in seven publications (21.9%) and directly measured in three (9.4%). Only one publication examined this competency within a pathway involving adolescent substance-use problems [19]. Cultural adaptation at the program level alone was not classified as evidence of parental competency; PC11 was assigned when an observable or potentially developable parenting practice, such as racial or cultural socialization, reinforcement of cultural identity, culturally grounded kinship roles, or family responses to discrimination, was identified.

Overall, parental competencies were more frequently incorporated as intervention targets than directly measured, and substantially fewer publications examined their relationships with adolescent substance-use outcomes. This pattern was evident even for the most frequently represented domains. In addition, some publications assessed multiple competencies using broader composite constructs, such as family relationships or positive parenting, which limited the ability to distinguish the contribution of individual competency domains.

### 3.4. Measurement Approaches and Informants

Considerable heterogeneity was observed in the measurement of parental competencies. No publication used a single comprehensive instrument designed to assess parental competency for adolescent substance use prevention across all 11 identified domains. Instead, most studies used domain-specific measures of communication, monitoring, discipline, family rules, relationship quality, parental support, or self-efficacy. Some publications combined several parenting constructs into broader measures of family relationships, positive parenting, family functioning, or family management [13,14,17,25]. Although these composite measures captured multiple dimensions of parenting, they limited the ability to distinguish individual competency domains.

Parent or caregiver self-report was the predominant measurement approach, particularly in intervention studies. Parents reported communication practices, monitoring behaviors, disciplinary consistency, family rules, relationship quality, parenting self-efficacy, and perceived influence over adolescent substance use [9,10,11,12,15,16,17,20,22,23,24,26,27,28,29]. The substance specificity of these measures varied. Some assessed general parent–adolescent communication or family functioning [23,26,27,28,29,30], whereas others specifically assessed communication about alcohol, tobacco, or other drugs, non-use expectations, and substance-related family rules [10,15,16,22,24,31,32].

Adolescent reports were also frequently used to assess parental behaviors, including monitoring, parental acceptance or disapproval of substance use, communication, emotional support, family involvement, relationship quality, and substance-related rules [9,13,15,16,17,22,24,25,31,32,33,36,38,39]. Several studies collected data from both parents and adolescents, allowing parental competencies to be examined from multiple perspectives [9,13,15,16,17,22,24,25,27,28,31]. However, parent and adolescent reports were not consistently analyzed separately, and some were incorporated into broader composite measures. Differences in parental competencies between mothers and fathers could not be systematically evaluated. Although some interventions specifically involved mothers [13,15,16], the broader literature generally reported parents or caregivers collectively and did not consistently provide competency findings stratified by parent gender. Consequently, the available evidence did not permit a reliable comparison of mother- and father-specific competency patterns.

Observational approaches were less common than questionnaire-based assessments. These included direct assessment of parental warmth and sensitivity during parent–child interactions [36] and videotaped family communication and monitoring tasks [17,22]. Recorded program sessions were also used to assess facilitator delivery and caregiver participation [20], although these primarily provided implementation evidence rather than direct assessment of parents’ everyday preventive competencies. Overall, direct behavioral observation of parental prevention competencies was limited.

Measurement approaches also varied within individual competency domains. Communication was operationalized through open communication, problem communication, communication frequency, listening, and substance-specific discussions [10,13,15,16,22,24,26,31,32]. Monitoring measures included parental knowledge of adolescents’ whereabouts, parental solicitation, behavioral control, adolescent disclosure, and supervision [9,15,16,22,25,29,31,33,36]. Relational bonding and family involvement were assessed through constructs such as warmth, closeness, emotional support, shared activities, cohesion, and instrumental support [9,13,14,15,16,23,27,28,29,31,32,36,38,39].

Prevention-specific measures were comparatively uncommon. Substance-related parental knowledge was directly measured through a drug-knowledge questionnaire in Reference [23], while prevention self-efficacy was assessed through parenting self-efficacy, Health Promotion Model self-efficacy, or perceived parental influence over adolescent substance use [11,12,29]. Prevention planning was directly assessed through commitment to a plan of action in Reference [11]. No direct measure of parents’ information- and resource-seeking competency was identified. Culturally responsive parenting was measured primarily through racial or cultural socialization constructs [9,19,20], whereas culturally grounded kinship and family practices were more often represented as intervention content than assessed using standardized measures [8,30]. The more abstract domains identified in this review may require particularly behavior-specific operationalization in future measurement. PC10 (Information and Resource Seeking) could be assessed through parents’ reported or demonstrated ability to actively seek credible prevention information, identify appropriate professional or community resources, navigate available services, initiate help-seeking or referrals when concerns arise, and use obtained information to inform preventive parenting decisions. This distinguishes PC10 from PC1 (Substance-Related Knowledge and Risk Awareness), which concerns what parents know or understand about substance-related risks rather than their ability to locate and mobilize information and resources. PC11 (Culturally Responsive Protective Parenting) could be assessed through culturally relevant indicators such as racial or cultural socialization practices, communication of cultural values and identity, preparation for discrimination or culturally specific risks, reinforcement of cultural pride and belonging, and mobilization of culturally grounded family, kinship, or community supports. These indicators distinguish PC11 from more general prevention communication (PC2) and relational bonding and family involvement (PC9) by focusing specifically on the intentional use of cultural and contextual resources as protective parenting practices.

The timing of parental competency measurement also varied. Some intervention studies assessed competencies only at immediate post-intervention [10,11,15,23,25,26,28,29,30], whereas others included follow-up assessments ranging from several months to two years [9,12,13,16,17,18,19,20,22,24,27,33,37]. A smaller group of publications examined parental competencies as mediators or developmental predictors of later substance-use outcomes [13,14,19,31,32,36,38,39]. Overall, measurement was characterized by substantial variation in construct definitions, substance specificity, informants, assessment timing, and the use of individual versus composite parenting measures.

### 3.5. Associations with Adolescent Substance-Use Outcomes

Only eight of the 32 included publications (25.0%) statistically examined one or more parental competency domains in relation to an adolescent substance-use outcome [13,14,19,31,32,36,38,39]. Five were observational studies [31,32,36,38,39], whereas three involved secondary or mediation analyses of randomized family-based interventions [13,14,19]. Outcomes included alcohol use and drunkenness, cigarette use, marijuana use, prescription drug misuse, other drug use, substance-use intentions, and composite measures of substance use or substance-related problems. Owing to heterogeneity in study design, developmental timing, competency measurement, and substance-use outcomes, these findings were synthesized descriptively.

Relational bonding and family involvement (PC9) had the largest body of association evidence and was examined in seven publications [13,14,31,32,36,38,39]. Longitudinal evidence indicated that parental warmth, support, and involvement were incorporated into developmental pathways involving later monitoring, adolescent psychosocial competencies, and substance-use outcomes [36,38,39]. Cross-sectional studies also examined relationship quality and family involvement in relation to adolescent substance use [31,32], although the temporal direction of these relationships could not be established.

Prevention communication (PC2) was examined in four publications [13,14,31,32], and monitoring and supervision (PC3) in three [13,31,36]. However, these competencies were frequently incorporated into broader constructs rather than analyzed independently. In the web-based mother–daughter prevention trial, communication, closeness, and parental monitoring were combined into a family-relationship mediator associated with adolescent self-efficacy and subsequent alcohol and marijuana use and substance-use intentions [13]. Similarly, the New Beginnings Program examined a positive-parenting construct encompassing relationship quality, communication, and effective discipline within developmental pathways involving later alcohol and marijuana use [14]. Consequently, the independent contributions of individual competency domains could not be determined in these analyses.

Evidence for the remaining behavioral competency domains was limited. Rules, boundaries, and expectations (PC4) were examined in relation to adolescent substance use in one publication [31], while consistent discipline and behavior management (PC5) was included within the broader positive-parenting construct in one publication [14]. Parental role modeling and family norms (PC7) were examined in one developmental study [36], in which parental acceptance of underage drinking was incorporated into pathways involving later adolescent substance use.

Culturally responsive protective parenting (PC11) was examined in one intervention-related pathway analysis [19]. In the Strong African American Families trial, intervention-related increases in parental racial socialization were associated with greater Black pride among adolescents, which was subsequently related to better psychological functioning and fewer substance-use problems and other adverse outcomes. Because this pathway involved multiple sequential mediators, it should not be interpreted as evidence of a direct effect of racial socialization on adolescent substance use.

No publication statistically examined substance-related knowledge and risk awareness (PC1), parental prevention self-efficacy (PC6), prevention planning and commitment (PC8), or information and resource seeking (PC10) in relation to an adolescent substance-use outcome. Overall, association evidence was concentrated on relational bonding, communication, and monitoring, with substantially less evidence for the other competency domains. The frequent use of composite parenting constructs further limited identification of the specific parental competencies associated with adolescent substance-use outcomes.

## 4. Discussion

### 4.1. Principal Findings

**Figure 2:**
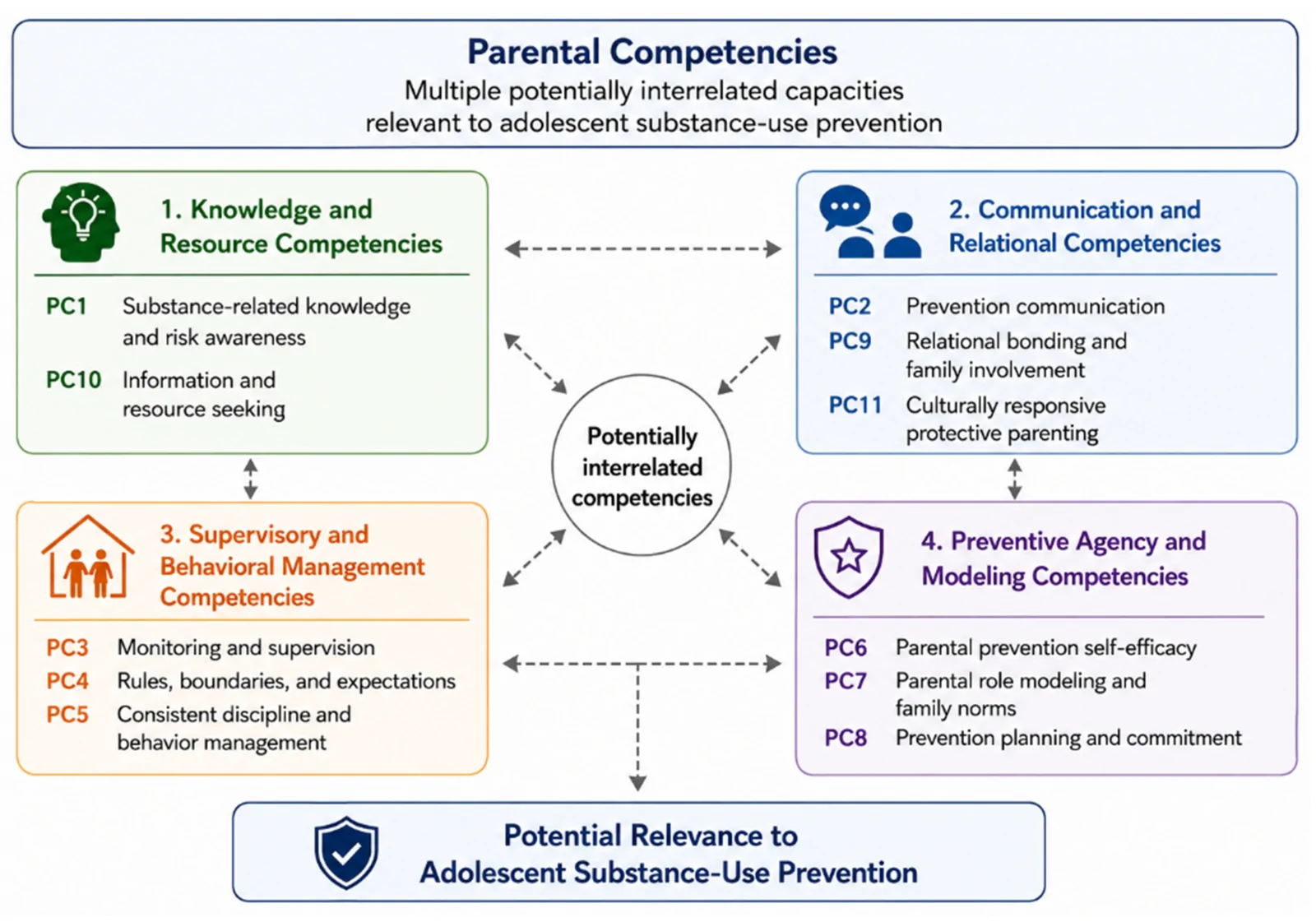
Conceptual Model of Parental Competencies Relevant to Adolescent Substance-Use Prevention. Note. The 11 parental competency domains identified in this scoping review were conceptually organized into four broader, potentially interrelated categories. The dashed arrows indicate potential relationships among competency categories and their relevance to adolescent substance-use prevention; they do not imply causal effects.

This scoping review showed that parental competency for adolescent substance use prevention is a multidimensional construct extending beyond general parenting ability. The identified literature primarily conceptualized preventive parenting through relational bonding, communication, monitoring, rule setting, and behavior management. These competencies were rarely presented as isolated capacities; instead, they functioned as interconnected family processes within broader developmental and relational contexts [13,14,36]. To facilitate conceptual interpretation of the 11 identified parental competency domains, we organized them into four broader, potentially interrelated categories: knowledge and resource competencies (PC1 and PC10), communication and relational competencies (PC2, PC9, and PC11), supervisory and behavioral management competencies (PC3-PC5), and preventive agency and modeling competencies (PC6-PC8) (Figure 2). This organization represents a conceptual synthesis of the mapped domains rather than an empirically validated factor structure. The categories are presented as potentially interrelated and relevant to adolescent substance-use prevention; however, the proposed relationships should not be interpreted as causal.

Relational bonding appears to provide an important foundation for other preventive parenting behaviors. Warmth, emotional support, and parent–adolescent closeness may encourage adolescents to disclose their activities and concerns, thereby allowing parents to monitor emerging risks and initiate preventive conversations. Longitudinal findings on nurturant parenting and parental support are consistent with this relational interpretation [38,39], suggesting that monitoring may function differently when it develops through trust and voluntary disclosure rather than relying primarily on parental surveillance or behavioral control. Communication was a central component of most parent- and family-focused approaches, but its conceptualization varied considerably. General parent–adolescent communication and substance-specific prevention communication represent related but distinct constructs and should not be treated as interchangeable [22,24,32]. A generally supportive relationship may facilitate prevention communication, but it does not necessarily indicate that parents can discuss substances accurately, confidently, and in a developmentally appropriate manner.

Similar conceptual ambiguity was evident for monitoring, rules, and discipline. Monitoring, rules, and discipline encompassed conceptually different practices, ranging from parental knowledge, solicitation, adolescent disclosure, and supportive supervision to behavioral control and more controlling forms of parenting [22,31,36]. Future research should distinguish protective structure from intrusive monitoring or punitive control because these practices may have different implications for family relationships and adolescent behavior.

The gap between intervention targeting and direct empirical evaluation has an important interpretive implication: changes in parenting outcomes do not necessarily establish that a specific competency accounts for subsequent adolescent substance-use outcomes, particularly when longer-term pathways are assessed using broad family-relationship or positive-parenting composites [10,11,12,13,14]. Such composites reflect the interconnected nature of family functioning but make it difficult to identify which specific parental competencies should be prioritized in interventions or incorporated into measurement instruments.

The limited assessment of prevention-specific cognitive and preparatory competencies suggests that general parenting competence alone may not adequately capture parents’ readiness to respond to emerging substance-related risks. This limitation is particularly relevant given the changing landscape of adolescent substance use, including electronic cigarettes, cannabis products, prescription drug misuse, and opioids. Substance-specific knowledge, judgment, and response preparedness therefore warrant greater attention in future measurement and intervention development.

Culturally responsive parenting emerged as a distinct but underexamined competency. Importantly, cultural adaptation of intervention language or materials should be distinguished from culturally responsive parenting itself, which involves observable practices such as racial socialization, cultural identity reinforcement, and culturally grounded kinship engagement [8,9,19]. This distinction is important for avoiding the assumption that a culturally adapted program automatically develops culturally responsive protective parenting.

Overall, these findings support conceptualizing parental competency as an integrated but differentiated set of capacities whose specific roles require more precise measurement and evaluation. This conceptualization may provide a clearer basis for future instrument development and competency-focused prevention interventions.

### 4.2. Parental Competencies as Modifiable Protective Factors

The findings of this review support interpreting parental competencies as potentially modifiable family-level protective factors rather than as fixed parental characteristics. Developmental evidence suggests that these competencies may operate through both preventive parenting behaviors and adolescent developmental processes, including monitoring, self-regulation, emotional adjustment, and social competence [36].

The included studies indicate that parental competencies function within a broader family system. Communication, monitoring, rules, discipline, and relational bonding are likely to influence one another rather than act independently. For example, supportive parent–adolescent relationships may facilitate voluntary disclosure and create a relational context in which guidance and family rules are more readily accepted. Intervention-mediated pathways involving family relationships and adolescent self-efficacy provide support for this interconnected process [13].

Developmental pathway findings further suggest that parental competencies may be linked to later substance-use outcomes through intermediate changes in adolescent competencies, emotional or behavioral adjustment, social anxiety, and refusal confidence [14,38]. Accordingly, evaluation of parental competencies should consider relevant intermediate developmental outcomes rather than focusing exclusively on immediate substance-use outcomes.

Among adolescents experiencing elevated individual or contextual risks, including emotional or behavioral difficulties, foster-care placement, or legal-system involvement, preventive parenting may require capacities beyond routine supervision and communication [22,27,28]. These may include recognizing vulnerability, responding without escalating conflict, coordinating with professionals, and maintaining supportive relationships despite instability or adversity.

The relevance and expression of parental competencies may also vary developmentally. Existing developmental evidence suggests that direct supervision may be more salient earlier in adolescence, whereas communication, negotiated expectations, and supportive involvement may become increasingly important as autonomy increases; supportive family processes may remain relevant into the transition to emerging adulthood [37]. However, these developmental patterns could not be directly compared across competency domains in the present review. Competency-based prevention should therefore be developmentally responsive rather than assuming that the same parental behaviors operate uniformly across ages 9–19 years.

Cultural and structural contexts further shape how parental competencies are expressed. Across culturally specific interventions, racial or cultural socialization, identity reinforcement, kinship and intergenerational relationships, and culturally grounded communication emerged as potentially relevant protective parenting processes [9,19,30]. These findings suggest that culturally responsive competencies should be understood within the relational and community contexts in which they are enacted rather than as supplementary adaptations to a standard parenting model.

Nevertheless, inclusion of a parental competency in a prevention program should not itself be interpreted as evidence of a protective effect. Moreover, the same parental behavior may have different implications depending on its intensity and relational context. Monitoring can provide protection when it reflects involvement and appropriate supervision but may become intrusive when implemented as excessive control. Likewise, family rules may provide structure when clearly communicated and consistently applied but may undermine trust when enforced harshly or without developmentally appropriate autonomy.

Accordingly, parental competencies may be best conceptualized as context-sensitive capacities involving not only what parents do but also how, when, and under what circumstances they act. Knowledge, skills, judgment, self-efficacy, and observable behavior should therefore be considered together. In this sense, parental competency is conceptually distinct from parenting style and family functioning. Parenting style generally refers to the broader and relatively stable relational or emotional climate in which parenting behaviors occur, whereas competency refers to specific, potentially developable capacities that parents can mobilize in response to prevention-related demands. Family functioning, in contrast, reflects broader characteristics of the family system, such as cohesion, communication, organization, and relational processes, and may involve multiple family members rather than parental capacities alone. Thus, although these constructs may overlap empirically, parental competency is distinguished by its focus on identifiable knowledge, skills, judgment, self-efficacy, and behaviors that can potentially be assessed and strengthened. This interpretation avoids reducing parental competency to either general parenting style or isolated preventive behavior and provides a stronger conceptual foundation for future measurement and intervention development.

### 4.3. Implications for Measurement and Instrument Development

The findings indicate a need for a multidimensional instrument specifically designed to assess parental competency for adolescent substance use prevention. Existing studies generally measured individual parenting constructs, such as communication, monitoring, discipline, relationship quality, or parental influence, rather than an integrated set of prevention competencies. For example, the Life Skills Training Parent Program assessed multiple parenting domains separately [10], providing domain-specific information but not capturing how these competencies may operate together in responding to substance-related risks.

Conversely, some studies used broad measures of family functioning or positive parenting. Broad measures used in studies such as Staying Connected with Your Teen and family-based prevention counseling encompassed multiple related parenting and family processes [17,25]. Composite measures may be useful for representing an overall family process, but they make it difficult to identify which specific parental competency is limited, responsive to intervention, or associated with adolescent substance use. A future instrument could therefore retain distinct subdomains while also permitting calculation of an overall competency score if supported psychometrically.

Construct definitions require particular attention. General parent–adolescent communication should be distinguished from prevention-specific communication. A parent may be emotionally available and communicate openly while lacking the knowledge or confidence required to discuss substances. The differing communication measures used in Familias and TALK further illustrate the need to distinguish general family communication from substance-specific prevention communication [24,26]. For assessment purposes, PC2 (Prevention Communication) and PC9 (Relational Bonding and Family Involvement) should therefore be distinguished according to the content and function of the parental behavior being assessed. PC2 specifically concerns explicit prevention-oriented communication about substance-related risks and consequences, parental expectations regarding non-use, and strategies for responding to peer pressure or seeking help. In contrast, PC9 concerns the broader relational foundation of warmth, trust, emotional availability, and ongoing family involvement, regardless of whether substance use is explicitly discussed. Accordingly, behavioral indicators for PC2 could include discussing substance-related risks and consequences, clearly communicating expectations regarding non-use, and discussing how an adolescent could respond to peer offers of substances or seek help when needed. Behavioral indicators for PC9 could include listening attentively when an adolescent expresses concerns, being emotionally available, spending meaningful time together, and maintaining a relationship in which the adolescent feels comfortable seeking parental support. These examples are intended as behavioral anchors for future instrument development rather than as validated questionnaire items.

Monitoring also requires greater conceptual precision. Existing measures variously assessed parental knowledge, supervision, solicitation, control, and adolescent disclosure. Because these indicators may reflect different parental behaviors and family processes, an integrated measure should distinguish parents’ active efforts to remain informed from knowledge obtained through voluntary adolescent disclosure. It should also differentiate developmentally appropriate supervision from intrusive control. Behaviorally anchored items, such as knowing an adolescent’s companions or checking agreed-upon plans, may be more interpretable than general items asking whether a parent “monitors” the adolescent.

Substance-specific knowledge and judgment should be included as separate measurement domains. The direct assessment of parental drug knowledge in Reference [23] illustrates one approach to operationalizing this domain, but future measurement should extend beyond recognition of substance names or health effects. Parents may also need to recognize behavioral warning signs, understand age-specific vulnerability, distinguish experimentation from more concerning patterns, evaluate the credibility of prevention information, and determine when professional assessment is required. Because patterns of alcohol, tobacco, cannabis, prescription drug, and illicit-drug use differ, an instrument should include both general prevention knowledge and substance-specific content.

Prevention self-efficacy, planning, and resource seeking require further development. Existing measures illustrate variation in the operationalization of parenting efficacy, preventive action, and perceived parental influence [11,12], but these constructs should not be treated as equivalent. Self-efficacy concerns whether parents believe they can perform a preventive behavior, whereas planning concerns whether they have selected specific actions, and resource seeking concerns whether they can obtain appropriate information or assistance. These competencies should therefore be represented by separate subscales rather than combined into a general confidence score.

Future items should emphasize observable or behaviorally interpretable capacities rather than global perceptions of parenting competence. Findings from the web-based mother–daughter intervention and its follow-up illustrate the value of assessing behaviorally interpretable competencies and their maintenance over time [15,16].

Reporter selection is another important consideration. Parent and adolescent reports may provide complementary information: parent reports can capture internal capacities such as knowledge, confidence, and planning, whereas adolescent reports can capture how relational and behavioral competencies are experienced. When feasible, parallel parent and adolescent versions could be developed for observable relational and behavioral domains, while parent-only items could assess internal knowledge, judgment, self-efficacy, and resource-navigation capacities.

Measurement should also account for developmental variation in the relevance and interpretation of competency indicators across adolescence. An instrument covering ages 9–19 should therefore be tested for measurement invariance or differential item functioning across developmental stages rather than assuming that identical behaviors have the same meaning at every age.

Cultural relevance should be established without treating culture as a demographic adjustment alone. Culturally responsive items should assess observable parental practices, such as reinforcing cultural identity, using culturally grounded family or kinship resources, and preparing adolescents to respond to discrimination or culturally specific risks. Because culture-specific competencies should not be assumed to apply uniformly across populations, a core instrument may combine broadly applicable domains with culturally adaptable modules evaluated within relevant populations.

Finally, instrument development should preserve the distinction between competency and outcome. Adolescent substance-use and broader family outcomes should not be incorporated into the competency construct, while parental substance use, socioeconomic adversity, and family instability should be treated as antecedent or contextual variables rather than competency indicators. Maintaining this distinction would allow future research to test whether parental competencies mediate or moderate relationships between family adversity, intervention exposure, and adolescent substance-use outcomes.

From a short-term instrument-development perspective, PC2 (Prevention Communication), PC4 (Rules, Boundaries, and Expectations), and PC5 (Consistent Discipline and Behavior Management) may warrant particular attention. These domains were repeatedly incorporated as intervention targets but were substantially less often assessed as distinct parental competencies, indicating clear target-to-measurement gaps. PC2 showed the largest absolute gap, being targeted in 26 publications but directly measured in 14, while PC4 was targeted in 16 but measured in six, and PC5 was targeted in 13 but measured in six. Their relatively behaviorally interpretable content may also facilitate the development of specific, observable indicators suitable for short-term measurement development. This prioritization reflects the combination of representation as intervention targets, measurement gaps, and feasibility of behavioral operationalization and should not be interpreted as evidence that these domains are more important or more effective than the other identified competencies. Less frequently measured domains, including prevention-specific knowledge, self-efficacy, planning, and resource seeking, remain important for the longer-term development of a comprehensive multidimensional instrument.

Overall, a future standardized measure should balance domain specificity with the multidimensional nature of parental competency. Such an instrument would permit researchers and practitioners to identify specific parental strengths and needs, evaluate change following intervention, and determine which competencies are prospectively associated with reduced adolescent substance use.

### 4.4. Implications for Prevention Interventions and Practice

The competency domains identified in this review provide a practical framework for designing and refining parent- and family-focused substance use prevention. Rather than delivering the same general parenting curriculum to all families, interventions could begin with an assessment of parents’ existing strengths and needs. Intervention content could then be tailored to specific needs in areas such as substance-related knowledge, communication, relational engagement, monitoring, or behavioral guidance. A modular approach may therefore be more appropriate than assuming that every family requires equal training across all competency domains.

Relational engagement should be treated as a foundation for other prevention components. Communication, monitoring, and rule setting are unlikely to operate independently of the quality of the parent–adolescent relationship. Family-focused interventions combining relational and behavioral components [21,33] suggest that prevention programs should combine behavioral guidance with relational skills rather than presenting monitoring or discipline as isolated control strategies.

Interventions should also distinguish between universal competencies and competencies needed by families experiencing elevated risk or adversity. Evidence from foster-care and justice-involved populations illustrates that families facing elevated adversity may require additional capacities related to trust, continuity, emotional and behavioral challenges, and risk management [27,28]. Accordingly, selective prevention should retain core competencies while adapting the intensity, delivery method, and supporting services to the family’s circumstances.

Developmental timing is equally important. Intervention priorities may shift from supervision, rules, and early prevention communication toward negotiated expectations, autonomy-supportive communication, and continued supportive involvement as adolescents mature, with supportive caregiving remaining relevant into emerging adulthood [37]. Parent gender should also be considered in future competency-focused research and intervention development. Because the current evidence did not permit systematic comparisons between mothers and fathers, future studies should assess mother- and father-specific competencies using comparable measures and report findings stratified by parent gender where appropriate. Such research could clarify whether particular competency domains differ in their relevance or expression between mothers and fathers and provide an empirical basis for determining whether gender-sensitive adaptations of parent-focused prevention interventions are warranted. Programs should therefore specify not only which competencies are addressed but also how they are adapted to adolescents’ developmental level.

Flexible delivery may improve parents’ access to preventive support. The alternative delivery formats used in Parents Who Care [18] illustrate the potential value of flexible approaches when work schedules, transportation, childcare responsibilities, stigma, and geographic distance may limit participation in conventional group programs. Digital and self-administered approaches may increase accessibility, although they should retain opportunities for individualized feedback, clarification, and skills practice.

Program duration and intensity should be matched to the complexity of the targeted competencies. Brief education may be sufficient for increasing awareness, but developing communication, monitoring, and family problem-solving skills may require repeated practice and feedback. Findings linking greater session attendance with improvements in parenting outcomes further suggest that intervention dose may be relevant to competency development [29]. Nevertheless, increasing the number of sessions alone may not improve outcomes if the content is burdensome, poorly tailored, or difficult to implement.

Schools can serve as important access points for parent-focused prevention, particularly before adolescents develop established patterns of substance use. Combined family- and school-based approaches may reinforce prevention across adolescents’ primary social environments [34], although parent participation may present additional implementation challenges. Programs should therefore minimize attendance barriers, provide remote or asynchronous alternatives, and clearly communicate how parent involvement contributes to adolescent health rather than presenting it as remediation for inadequate parenting.

Community engagement is particularly important when adapting interventions for culturally or socially diverse families. Community-engaged approaches illustrate how stakeholder participation, cultural acceptability, and scientific evidence can jointly inform intervention content and delivery [9,35]. Cultural adaptation should extend beyond translation or surface-level modification to consider culturally grounded resources such as identity, family and kinship relationships, community participation, and culturally relevant social support [30]. Such components should be developed with the relevant communities and evaluated within those populations rather than generalized across cultural contexts.

Implementation quality must also be considered when interpreting program outcomes. A theoretically appropriate competency component may have little effect if families do not attend, facilitators omit key material, or skills are not practiced outside intervention sessions. Evidence from SAAF further highlights the relevance of fidelity, facilitator adaptation, and participant engagement to the delivery and use of parenting content [20]. Future trials should therefore assess not only whether a competency was included in the curriculum but also whether parents received, practiced, and applied it.

In clinical, school-health, and community practice, the identified domains could be used to guide brief parental assessment and individualized support. Nurses and other health professionals could identify specific needs in substance-related knowledge, communication, supervision, expectations, or resource navigation and link families to appropriate education, counseling, family intervention, or community resources. Such assessment should be strengths-based and should avoid attributing adolescent substance use solely to parental behavior.

Overall, effective parent-focused prevention should be developmentally responsive, culturally grounded, accessible, and sufficiently flexible to address different family needs. The competency framework may support this approach by clarifying the parental capacities targeted by interventions and increasing precision in component selection, implementation assessment, and evaluation of competency change.

### 4.5. Strengths and Limitations

This scoping review has several strengths. First, it provides a systematic mapping of parental competencies relevant to adolescent substance use prevention across a heterogeneous body of literature that included intervention, observational, program-development, cultural-adaptation, feasibility, and implementation studies. Rather than focusing solely on whether parent- or family-based interventions reduced substance use, the review examined the specific parental capacities represented within these studies. This approach enabled a more differentiated characterization of the parental capacities represented across the literature.

Second, the review distinguished among competencies that were targeted as intervention content, directly measured as parental capacities, and statistically examined in relation to adolescent substance-use outcomes. This evidence-role distinction, together with the separation of potentially modifiable parental competencies from parental risk characteristics, contextual conditions, adolescent competencies, and intervention outcomes, provides greater conceptual clarity for future competency-based measurement and intervention development.

Several limitations should also be considered. First, the included literature was heterogeneous in study design, population, intervention characteristics, measurement approaches, follow-up periods, and substance-use outcomes. The review therefore aimed to map the available evidence rather than quantitatively estimate the effects of individual parental competencies. The frequency with which a competency appeared in the literature should not be interpreted as evidence of its relative importance, effectiveness, or causal influence on adolescent substance use.

Second, the publication was used as the primary unit of descriptive mapping. Some publications originated from the same underlying trial or prevention program and were retained separately because they contributed distinct information regarding intervention content, measurement, mechanisms, implementation, or follow-up. Although shared study origins were considered in interpretation, publication-level frequencies may give greater representation to competencies examined in programs generating multiple reports and should therefore be understood as representation across publications rather than as counts of independent supporting studies.

Third, competency classification required conceptual interpretation because the terminology and operationalization of parenting constructs varied substantially across studies. Because similarly labeled parenting constructs sometimes encompassed overlapping behaviors, some degree of conceptual overlap among domains remained despite the use of predefined operational criteria. In addition, broad composite parenting measures limited the ability to determine which specific competency accounted for observed associations with adolescent substance-use outcomes. An additional measurement limitation concerns the predominant reliance on parent or caregiver self-report. Self-reported competencies may be affected by social desirability, recall bias, and discrepancies between parents’ perceptions of their own practices and how those practices are experienced by adolescents. Reliance on a single informant may therefore provide an incomplete representation of parental competency, particularly for observable relational and behavioral domains. Future research should use multimethod and multi-informant assessment strategies that combine parent reports with adolescent reports and, where feasible, direct or structured observational measures of parent–adolescent interactions. Such triangulation may provide a more valid and comprehensive assessment by capturing both parents’ internal capacities, such as knowledge and self-efficacy, and the observable enactment and adolescent experience of preventive parenting competencies.

Fourth, an additional limitation concerns developmental-stage comparisons. Although adolescent age ranges were extracted from the included publications, a reliable subgroup analysis across early, middle, and late adolescence was not feasible because many studies included broad or overlapping age ranges and did not report parental competency findings separately by developmental stage. Assigning such studies to a single developmental category based only on their overall age range could therefore have introduced misclassification. Future studies should report competency findings using developmentally stratified age groups to clarify whether the relevance or expression of specific parental competencies differs across adolescence. Similarly, mother–father comparisons were not undertaken in the present review, and such comparisons could not be reliably conducted retrospectively because most included publications did not report parental competency findings separately by parent gender. Accordingly, the present review could not determine whether specific competency domains differed systematically between mothers and fathers. This limitation highlights the need for future studies to report parent-gender-stratified competency data using comparable measures.

Fifth, the available evidence was insufficient to establish that individual parental competency domains independently prevent adolescent substance use. Accordingly, the identified domains should be interpreted as potentially modifiable protective factors represented in the literature rather than as competencies with established independent preventive effects.

Finally, the review included only peer-reviewed original research articles published in English. Relevant evidence reported in other languages, grey literature, dissertations, reports, or other non-peer-reviewed sources may therefore have been missed. In addition, although the search covered multiple international and Korean databases without geographical restrictions, the evidence base was concentrated in particular countries and cultural contexts. The transferability of the identified competency framework across different family structures, cultural groups, health systems, and socioeconomic contexts therefore requires further investigation.

## 5. Conclusions

This scoping review mapped parental competencies as potentially modifiable family-level protective factors for adolescent substance use prevention. Across 32 publications, 11 competency domains were identified, with relational bonding and family involvement, prevention communication, monitoring and supervision, and rules, boundaries, and expectations most frequently represented. However, parental competencies were more often incorporated as intervention targets than directly measured or statistically examined in relation to adolescent substance-use outcomes, highlighting an important gap between prevention content and empirical assessment.

The findings suggest that parental competency for substance use prevention is multidimensional, encompassing relational, communicative, supervisory, behavioral, cognitive, and culturally responsive capacities. Based on the gaps identified in this review, three practical and research priorities warrant particular emphasis. First, a standardized, multidimensional instrument should be developed and validated to assess specific parental competencies while accounting for developmental and cultural contexts. Second, prevention interventions should be designed and evaluated around clearly specified parental competencies, rather than relying primarily on generic parenting or broad family-functioning constructs, so that competency-specific changes can be assessed. Third, longitudinal studies should examine whether and how specific parental competencies, and changes in these competencies, mediate pathways to subsequent adolescent substance-use outcomes. Addressing these priorities would help strengthen the measurement, intervention specificity, and longitudinal evidence needed to advance competency-based approaches to adolescent substance-use prevention.

## Figures and Tables

**Table 1:** PCC framework of the scoping review.

PCC Element	Definition
Population	Parents or primary caregivers of adolescents aged 9–19 years, including studies in which parental competencies or parenting practices were reported by adolescents
Concept	Parental knowledge, skills, judgment, self-efficacy, and behaviors that are identifiable, measurable, or modifiable as family-level protective factors for preventing adolescent substance use
Context	Family and home settings, as well as parent-involved school, community, clinical, and digital prevention contexts, without geographical restrictions

Note. Substance use included alcohol, tobacco and nicotine products, cannabis, prescription drug misuse, inhalants, and other illicit or unspecified substances. Studies focused exclusively on the treatment or rehabilitation of established substance use disorders were outside the review scope. PCC = Population, Concept, and Context.

**Table 2:** Parental competency domains identified across the included publications (N = 32).

Code	Parental Competency Domain	Operational Definition	Representative Indicators	Publications, n (%)	Reference Numbers
PC1	Substance-related knowledge and risk awareness	Parents’ understanding of substances, developmental risks, warning signs, and prevention information.	Substance-related knowledge; recognition of risks or warning signs; prevention-related risk awareness	6 (18.8)	[10,11,12,22,23,24]
PC2	Prevention communication	Parents’ ability to initiate and maintain clear, developmentally appropriate, and bidirectional communication specifically concerning substance use, related risks, preventive expectations, and response strategies.	Substance-specific preventive conversations; discussion of substance-related risks and consequences; communication of non-use expectations; discussion of peer-pressure, refusal, and help-seeking strategies	28 (87.5)	[8,9,10,11,12,13,14,15,16,17,18,19,20,21,22,23,24,25,26,27,28,29,30,31,32,33,34,35]
PC3	Monitoring and supervision	Parents’ active awareness and supervision of adolescents’ whereabouts, peers, activities, and opportunities for substance exposure.	Monitoring whereabouts and peers; supervision; checking activities; parental knowledge derived from active monitoring	20 (62.5)	[8,9,10,12,13,15,16,17,18,19,21,22,25,26,29,31,33,34,35,36]
PC4	Rules, boundaries, and expectations	Parents’ establishment and communication of clear family rules, limits, and expectations related to substance use and associated behaviors.	Family rules; limit-setting; explicit expectations of non-use; stated consequences	17 (53.1)	[9,12,13,15,16,17,18,19,21,22,24,25,28,31,33,34,35]
PC5	Consistent discipline and behavior management	Parents’ consistent and constructive responses to adolescent behavior and violations of family expectations.	Consistent discipline; reinforcement; non-harsh consequences; behavior management and problem-solving	13 (40.6)	[9,10,12,14,17,18,19,20,21,25,27,29,34]
PC6	Parental prevention self-efficacy	Parents’ confidence in their ability to perform prevention-related parenting actions despite difficulties or barriers.	Confidence in discussing substance use, monitoring, setting limits, preventing use, or responding to risk	4 (12.5)	[11,12,22,29]
PC7	Parental role modeling and family norms	Parents’ modeling of non-use or healthy behavior and communication of protective family norms regarding substance use.	Non-use modeling; responsible behavior; parental disapproval of substance use; protective family norms	8 (25.0)	[10,12,17,18,33,35,36,37]
PC8	Prevention planning and commitment	Parents’ development of concrete plans, commitments, and response strategies for preventing or addressing adolescent substance use.	Prevention action plans; commitment to preventive action; preparedness; agreed response strategies	3 (9.4)	[11,22,24]
PC9	Relational bonding and family involvement	Parents’ maintenance of warmth, attachment, emotional availability, trust, and active involvement that provides a general relational foundation within which preventive parenting can occur.	Warmth and nurturance; emotional availability; parent–adolescent trust and closeness; attentive listening and supportive engagement; shared family activities; adolescent comfort in seeking parental support	29 (90.6)	[8,9,13,14,15,16,17,18,19,20,21,22,23,24,25,26,27,28,29,30,31,32,33,34,35,36,37,38,39]
PC10	Information and resource seeking	Parents’ ability to identify, seek, evaluate, and use credible prevention information, professional help, and community resources.	Active searching for credible substance-use prevention information; identifying appropriate professional or community services; knowing where and how to obtain help; navigating available prevention or treatment resources; initiating help-seeking or referrals when concerns arise; using obtained information or resources to inform preventive parenting decisions.	4 (12.5)	[8,10,22,24]
PC11	Culturally responsive protective parenting	Parents’ use of cultural identity, traditions, racial or ethnic socialization, and kinship resources as protective parenting practices.	Engaging in racial or cultural socialization; communicating cultural values, traditions, and identity; preparing adolescents to cope with discrimination or culturally specific risks; reinforcing cultural pride and belonging; incorporating culturally grounded family or kinship roles into preventive parenting; mobilizing culturally relevant family or community supports.	7 (21.9)	[8,9,19,20,21,30,37]

Note. Parental competency domains were not mutually exclusive, and each publication could be assigned to more than one domain. Frequencies represent the number of publications in which each competency domain was identified or represented in the included literature and should not be interpreted as evidence of its relative importance or effectiveness. Percentages were calculated using the 32 included publications as the denominator.

**Table 3:** Summary of Parental Competency Domains and Evidence Roles across the Included Publications (N = 32).

Code	Parental Competency Domain	Publications, n (%)	Targeted, n (%)	Measured, n (%)	Associated, n (%)
PC1	Substance-related knowledge and risk awareness	6 (18.8)	6 (18.8)	1 (3.1)	0 (0.0)
PC2	Prevention communication	28 (87.5)	26 (81.3)	14 (43.8)	4 (12.5)
PC3	Monitoring and supervision	20 (62.5)	18 (56.3)	12 (37.5)	3 (9.4)
PC4	Rules, boundaries, and expectations	17 (53.1)	16 (50.0)	6 (18.8)	1 (3.1)
PC5	Consistent discipline and behavior management	13 (40.6)	13 (40.6)	6 (18.8)	1 (3.1)
PC6	Parental prevention self-efficacy	4 (12.5)	3 (9.4)	3 (9.4)	0 (0.0)
PC7	Parental role modeling and family norms	8 (25.0)	7 (21.9)	2 (6.3)	1 (3.1)
PC8	Prevention planning and commitment	3 (9.4)	3 (9.4)	1 (3.1)	0 (0.0)
PC9	Relational bonding and family involvement	29 (90.6)	24 (75.0)	18 (56.3)	7 (21.9)
PC10	Information and resource seeking	4 (12.5)	4 (12.5)	0 (0.0)	0 (0.0)
PC11	Culturally responsive protective parenting	7 (21.9)	7 (21.9)	3 (9.4)	1 (3.1)

Note. Competency domains were not mutually exclusive, and each publication could contribute to more than one domain and evidence role. Targeted indicates that the competency was explicitly incorporated as intervention content; measured indicates that it was directly assessed as a parent or caregiver construct; and associated indicates that it was statistically examined in relation to an adolescent substance-use outcome. Classification as associated does not necessarily indicate statistical significance, causality, or intervention effectiveness. Percentages were calculated using the 32 included publications as the denominator.

## Data Availability

No new data were created or analyzed in this study. Data sharing is not applicable to this article. All data supporting the findings of this scoping review are available within the article and its Supplementary Materials.

## Supplementary Materials

The following supporting information can be downloaded at: https://www.mdpi.com/article/10.3390/children13091193/s1, Table S1: Preferred Reporting Items for Systematic Reviews and Meta-Analyses Extension for Scoping Reviews (PRISMA-ScR) Checklist; Table S2: Search Strategy (PubMed); Table S3: Characteristics of the Included Publications (N = 32); Table S4. Evidence Mapping of Parental Competency Domains across the Included Publications (N = 32). Reference [40] is cited in the supplementary materials.
