# Supplementary material for: Parental Competencies as Familial Protective Factors in Adolescent Substance Use Prevention: A Scoping Review
Source: Children (Basel). 2026 Sep 4;13(9):1193. doi: 10.3390/children13091193 (PMC13605026; doi:10.3390/children13091193)
Supplement: Supplementary file 1 [file children-13-01193-s001.zip › children-4527030-supplementary.pdf]

**Supplementary Table S1.** Preferred Reporting Items for Systematic reviews and Meta-Analyses extension for Scoping Reviews (PRISMA-ScR) Checklist.

| SECTION | ITEM | PRISMA-ScR CHECKLIST ITEM | REPORTED ON PAGE # |
|---|---|---|---|
| **TITLE** | | | |
| Title | 1 | Identify the report as a scoping review. | 1 |
| **ABSTRACT** | | | |
| Structured summary | 2 | Provide a structured summary that includes (as applicable): background, objectives, eligibility criteria, sources of evidence, charting methods, results, and conclusions that relate to the review questions and objectives. | 1 |
| **INTRODUCTION** | | | |
| Rationale | 3 | Describe the rationale for the review in the context of what is already known. Explain why the review questions/objectives lend themselves to a scoping review approach. | 2-3 |
| Objectives | 4 | Provide an explicit statement of the questions and objectives being addressed with reference to their key elements (e.g., population or participants, concepts, and context) or other relevant key elements used to conceptualize the review questions and/or objectives. | 4 |
| **METHODS** | | | |
| Protocol and registration | 5 | Indicate whether a review protocol exists; state if and where it can be accessed (e.g., a Web address); and if available, provide registration information, including the registration number. | 4 |
| Eligibility criteria | 6 | Specify characteristics of the sources of evidence used as eligibility criteria (e.g., years considered, language, and publication status), and provide a rationale. | 4-5 |
| Information sources | 7 | Describe all information sources in the search (e.g., databases with dates of coverage and contact with authors to identify additional sources), as well as the date the most recent search was executed. | 5 |

| SECTION | ITEM | PRISMA-ScR CHECKLIST ITEM | REPORTED ON PAGE # |
|---|---|---|---|
| Search | 8 | Present the full electronic search strategy for a t least 1 database, including any limits used, s uch that it could be repeated. | 5-6; Supple mentary Tab le S2 |
| Selection of sourc es of evidence | 9 | State the process for selecting sources of evid ence (i.e., screening and eligibility) included in the scoping review. | 6-7 |
| Data charting process | 10 | Describe the methods of charting data from th e included sources of evidence (e.g., calibrated forms or forms that have been tested by the team before their use, and whether data charti ng was done independently or in duplicate) an d any processes for obtaining and confirming d ata from investigators. | 7 |
| Data items | 11 | List and define all variables for which data wer e sought and any assumptions and simplificatio ns made. | 7 |
| Critical appraisal of individual sources of evidence | 12 | If done, provide a rationale for conducting a cr itical appraisal of included sources of evidence; describe the methods used and how this infor mation was used in any data synthesis (if appr opriate). | N/A |
| Synthesis of results | 13 | Describe the methods of handling and summari zing the data that were charted. | 7-8 |
| **RESULTS** | | | |
| Selection of sources of evidence | 14 | Give numbers of sources of evidence screened , assessed for eligibility, and included in the re view, with reasons for exclusions at each stag e, ideally using a flow diagram. | 6-7; Figure 1 |
| Characteristics of sources of evidence | 15 | For each source of evidence, present character istics for which data were charted and provide the citations. | 8-9; Supple mentary Tab le S3 |
| Critical appraisal within sources of | 16 | If done, present data on critical appraisal of in cluded sources of evidence (see item 12). | N/A |

| SECTION | ITEM | PRISMA-ScR CHECKLIST ITEM | REPORTED ON PAGE # |
|---|---|---|---|
| evidence | | | |
| Results of individual sources of evidence | 17 | For each included source of evidence, present the relevant data that were charted that relate to the review questions and objectives. | 9-16; Tables 2 and 3; Supplementary Tables S3 and S4 |
| Synthesis of results | 18 | Summarize and/or present the charting results as they relate to the review questions and objectives. | 9–16; Tables 2 and 3 |
| **DISCUSSION** | | | |
| Summary of evidence | 19 | Summarize the main results (including an overview of concepts, themes, and types of evidence available), link to the review questions and objectives, and consider the relevance to key groups. | 16-23 |
| Limitations | 20 | Discuss the limitations of the scoping review process. | 23-24 |
| Conclusions | 21 | Provide a general interpretation of the results with respect to the review questions and objectives, as well as potential implications and/or next steps. | 24-25 |
| **FUNDING** | | | |
| Funding | 22 | Describe sources of funding for the included sources of evidence, as well as sources of funding for the scoping review. Describe the role of the funders of the scoping review. | 25 |

Source: Tricco, A.C.; Lillie, E.; Zarin, W.; O'Brien, K.K.; Colquhoun, H.; Levac, D.; et al. PRISMA Extension for Scoping Reviews (PRISMA-ScR): Checklist and Explanation. Ann. Intern. Med. 2018, 169, 467–473. https://doi.org/10.7326/M18-0850

**Supplementary Table S2.** Search Strategy (PubMed).

| No. | Search terms |
|---|---|
| 1 | “Adolescent” [MeSH] |
| 2 | Adolescent* OR Teen* OR Youth* OR "Young people" OR "Young person*" OR "Middle school student*" OR "High school student*" |
| 3 | 1 OR 2 |
| 4 | “Parents” [MeSH] |
| 5 | Parent* OR Caregiver* OR "Primary caregiver*" OR Mother* OR Father* OR Family* |
| 6 | 4 OR 5 |
| 7 | “Parenting” [MeSH] |
| 8 | "Parental competenc*" OR "Parenting competenc*" OR "Parenting skill*" OR "Parenting practice*" OR "Parenting behavio*" OR "Parental self-efficacy" OR "Parenting self-efficacy" OR "Parental monitoring" OR "Parental supervision" OR "Parent-child communication" OR "Parent-adolescent communication" OR "Parental support" OR "Parental involvement" OR "Parental rule*" OR "Family rule*" OR "Parental expectation*" OR "Parent-child relationship*" |
| 9 | 7 OR 8 |
| 10 | “Substance-Related Disorders”[MeSH] |
| 11 | "Substance use" OR "Substance abuse" OR "Substance misuse" OR Alcohol* OR Tobacco OR Smoking OR Nicotine OR Vaping OR "Electronic cigarette*" OR Cannabis OR Marijuana OR "Prescription drug misuse" OR Inhalant* OR "Illicit drug*" OR "Drug use" |
| 12 | 10 OR 11 |
| 13 | “Prevention and Control”[Subheading] OR “Primary Prevention”[MeSH] OR “Health Promotion”[MeSH] |
| 14 | Prevent* OR Intervention* OR "Preventive intervention*" OR "Prevention program*" OR "Family-based prevention" OR "Parent-based prevention" OR "Parent-focused prevention" OR "Substance use prevention" |
| 15 | 13 OR 14 |
| 16 | 3 AND 6 AND 9 AND 12 AND 15 |

**Supplementary Table S3.** Characteristics of the Included Publications (N = 32).

| Reference | Author (year), country | Study design | Participants and adolescent age | Program or parental factor | Intervention duration/dose or observation period | Follow-up period | Substance |
|---|---|---|---|---|---|---|---|
| [39] | Wills and Cleary (1996), United States | Three-wave longitudinal school-based observational study | Grade-specific samples of 1,702 in grade 7, 1,826 in grade 8, and 1,895 in grade 9; mean age 12.4 years at first assessment | Emotional and instrumental parental support | Annual classroom assessments across grades 7–9 | Two 1-year intervals | Tobacco, alcohol, heavy drinking, and cannabis |
| [25] | Hogue et al. (2002), United States | Randomized controlled trial | 124 high-risk African American youths and their caregivers; adolescents aged 11–14 years | Multidimensional family-based prevention counseling | Individually tailored 15–25 sessions delivered over 3–4 months, primarily in the | Immediate posttest; mean interval from pretest to posttest approximately 4 months | Alcohol, cigarettes, cannabis, and other drugs |

| | | | | | | | |
|---|---|---|---|---|---|---|---|
| | | | | | home | | |
| [34] | Spoth et al. (2002), United States | Cluster-randomized controlled trial involving 36 rural schools | 1,664 seventh-grade students at baseline; 1,372 retained for longitudinal analysis | Combined Life Skills Training and Strengthening Families Program 10–14, Life Skills Training alone, or control | Life Skills Training: 15 classroom sessions; Strengthening Families: seven weekly family sessions with separate 1-hour parent/youth sessions and a 1-hour joint session; four booster sessions in grade 8 | Substance-initiation outcomes 1 year after intervention posttest | Alcohol, cigarettes, and cannabis |
| [32] | Wills et al. (2003), United States | Cross-sectional path-analysis study | 297 African American adolescents; ages 12–14 years, mean | Parent–child relationship quality and direct family communication about substances | Not applicable; one-time community survey | No follow-up | Cigarettes, alcohol, heavy drinking, and illicit drugs |

| | | | | | | | |
|---|---|---|---|---|---|---|---|
| | | | 13.0 | | | | |
| [30] | Harvey and Hill (2004), United States | Three-year pre-experimental process and outcome evaluation with comparison data | 57 participating African American boys and 30 comparison youths; ages 11.5–14.5 years; parent data available for 12 at pretest and 10 at posttest | MAAT Africentric Adolescent and Family Rites of Passage Program | Each cohort participated for one school year; after-school activities 3 hours/day, 3 days/week, plus family empowerment activities and individual/family counseling | Pretest and posttest within each annual cohort across the 3-year evaluation | Alcohol and other drugs; principal outcome was drug-risk knowledge |
| [23] | Crunkilton et al. (2005), United States | One-group pre-experimental pretest–posttest study | 49 Latino parents at baseline and 32 at posttest; children's exact ages NR | Proyecto Juventud, a culturally tailored family intervention | One-year family intervention, including 10 family camps and 13 family-support meetings held | Posttest approximately 12 months after baseline | Alcohol, tobacco, and other drugs |

| | | | | | | | |
|---|---|---|---|---|---|---|---|
| | | | | | approximately every 3–4 weeks | | |
| [12] | Riggs et al. (2006), United States | Parent-component analysis within a school-based prevention trial | 351 parents with baseline and 2-year data from eight schools; youths in grades 7–8 when the parent program began | Project STAR parent program: parent–school committee, parent skills training, and parent–child homework | Parent component implemented over one school year; one 2.5-hour skills workshop offered on two occasions, committee activities, and parent–child homework exercises | 2 years | Multiple or unspecified drugs |
| [18] | Haggerty et al. (2007), United | Randomized controlled trial | 331 Black and White families; adolescents in grade 8 at | Parents Who Care | Seven-session program; group format delivered weekly over 7 | Posttest, 12 months, and 24 months | Alcohol, tobacco, and other drugs |

| | | | | | | | |
|---|---|---|---|---|---|---|---|
| | States | | intervention | | weeks in 2–2.5-hour sessions or self-administered format completed within 10 weeks with weekly telephone support | | |
| [15] | Fang et al. (2010), United States | Randomized controlled trial | 108 Asian American mother–daughter dyads: intervention 56 and control 52; girls aged 10–14 years | Web-based mother–daughter substance-use prevention program | Nine weekly web-based sessions; mean intervention completion period approximately 6.25 months | Posttest approximately 1 year after baseline | Alcohol, cannabis, and prescription drug misuse |
| [10] | Griffin et al. (2011), United | Randomized controlled trial | 338 parents of middle-school students | Self-administered home-based Life Skills Training Parent Program | 15 home-based sessions designed for | Approximately 1 month postintervention and 1 year | Multiple or unspecified substances |

| | | | | | | | |
|---|---|---|---|---|---|---|---|
| | States | | | | completion over 15 weeks | | |
| [31] | Fang et al. (2011), United States | Cross-sectional correlational study | 135 Asian American mother–daughter dyads; girls aged 11–14 years, mean 13.2 | Maternal monitoring, communication, involvement, and family rules | Not applicable; one-time online survey | No follow-up | Alcohol, prescription drug misuse, and other drugs |
| [21] | Brody et al. (2012), United States | Randomized controlled trial with attention control | 502 Black families: intervention 252 and control 250; adolescents aged 16 years at baseline | Strong African American Families–Teen (SAAF–T) | Five weekly family meetings; 10 total hours | 22 months | Alcohol, tobacco, and cannabis, analyzed as a composite |
| [26] | Williams et al. (2012), United States | Cluster-randomized school-based trial | 393 predominantly Latino parents from nine middle schools; youths' mean age 12.2 | Familias Preparando la Nueva Generación, the parent component of keepin' it REAL | Eight weekly parent workshops; approximately 5–10 parents per group | Immediate posttest after the 8-week program | Tobacco, alcohol, and other drugs |

| | | | | | | | |
|---|---|---|---|---|---|---|---|
| | | | years | | | | |
| [37] | Brody et al. (2012), United States | Randomized controlled trial | 347 rural African American youths: intervention 174 and control 173; mean age 17.7 years | Adults in the Making | Six consecutive weekly meetings; concurrent 1-hour parent and youth sessions followed by a 1-hour joint session; 12 total hours | 6.4, 16.6, and 27.5 months after baseline | Alcohol and substance-use-related problems |
| [29] | Allen et al. (2013), United States | One-group pretest–posttest feasibility study using community-based participatory research | 83 immigrant Latino parents of adolescents aged 10–14 years | Padres Informados/Jóvenes Preparados | Eight Spanish-language parent classes, 2.5 hours per session; parents attended a mean of six sessions | Immediate posttest after the final session | Tobacco and other substances |
| [16] | Fang and | Two-year | 108 Asian | Web-based mother– | Nine web-based | 1 and 2 years | Alcohol, cannabis, and |

| | | | | | | | |
|---|---|---|---|---|---|---|---|
| | Schinke (2013), United States | follow-up of a randomized controlled trial | American mother–daughter dyads: intervention 56 and control 52; girls aged 10–14 years | daughter substance-use prevention program | modules followed by one annual booster session | | prescription drug misuse |
| [20] | Berkel et al. (2013), United States | Mixed-methods implementation and secondary outcome analysis of an RCT | SAAF efficacy-trial families with 11-year-old children; pretest sample included 157 intervention and 127 control families; implementation analyses focused on exposed intervention families | Strong African American Families (SAAF) | Seven consecutive weekly meetings; separate 1-hour caregiver and youth sessions followed by a 1-hour joint family session | Posttest began 3 months after program completion; approximately 7 months between pretest and posttest | Multiple substances; substance-use prevention was a program target rather than the primary outcome of this report |
| [13] | Fang and | Secondary | 108 Asian | Web-based mother– | Nine web-based | 1 and 2 years | Alcohol and cannabis; future |

| | | | | | | | |
|---|---|---|---|---|---|---|---|
| | Schinke (2014), United States | mediation analysis of a randomized controlled trial | American mother–daughter dyads; girls aged 10–14 years | daughter substance-use prevention program | modules completed jointly by mothers and daughters | | intention to use substances |
| [22] | Spirito et al. (2015), United States | Open pilot followed by pilot randomized controlled trial | 72 families: 5 open-pilot and 67 randomized families; adolescents aged 11–17 years | Family Check-Up, compared with psychoeducation | One-hour family assessment, 90–120-minute individualized feedback session, and eight mailed booster materials | 3 and 6 months | Alcohol, cigarettes, cannabis, and other drugs |
| [17] | Haggerty et al. (2015), United States | Long-term follow-up of a randomized controlled trial | 331 Black and White families; adolescents in grade 8 at intervention | Parents Who Care/Staying Connected with Your Teen | Seven-chapter program; group format delivered in seven weekly sessions of approximately 2–2.5 hours or | Earlier 24-month assessment and long-term assessment at age 20 | Alcohol, tobacco, cannabis, and other drugs |

| | | | | | | | |
|---|---|---|---|---|---|---|---|
| | | | | | self-administered format completed within 10 weeks with telephone support | | |
| [36] | Eiden et al. (2016), United States | Prospective longitudinal cohort study | 227 families: 125 families with paternal alcohol disorder and 102 comparison families | Parental warmth/sensitivity, monitoring, and acceptance of underage drinking | Repeated assessments from child age 12 months through late adolescence | Substance-use outcomes assessed at ages 15–19 years | Alcohol and cannabis; cigarettes and other drugs additionally assessed |
| [14] | Wolchik et al. (2016), United States | Fifteen-year follow-up and developmental cascade analysis of a randomized trial | 240 emerging adults; aged 9–12 years at the original intervention | New Beginnings Program for divorced families | Parent program comprising 11 group sessions of 1.75 hours and two individual sessions; some children concurrently | Posttest, 3 and 6 months, 6 years, and 15 years | Alcohol and cannabis |

| | | | | | | | |
|---|---|---|---|---|---|---|---|
| | | | | | received an 11-session child program | | |
| [38] | Weymouth et al. (2019), United States | Four-wave longitudinal observational study | 687 two-parent families; adolescents' mean age 11.3 years in grade 6 | Maternal and paternal nurturant-involved parenting | Four assessments from fall of grade 6 through grade 8 | Approximately 2 academic years | Alcohol intoxication, cigarettes, and cannabis |
| [9] | Whitesell et al. (2019), United States | Randomized fractional-factorial optimization trial embedded in community-engaged adaptation | 136 families enrolled; analytic sample 98 families, 137 adults, and 122 youths; youths aged 10–14 years, mean 11.5 | Thiwáhe Gluwáš'akapi, adapted from Strengthening Families Program 10–14 | Seven weekly family meetings; experimental adaptation components included tribal language, social-media engagement, and substance-use content | End-of-program assessment, within 2 months after completion, and approximately 6 months for eligible cohorts | Alcohol, cigarettes, and cannabis |
| [8] | Ivanich et | Comparative | Two Indigenous | Adaptations of Bii-Zin-Da- | Community- | No effectiveness follow-up | Primarily alcohol and other |

| | | | | | | | |
|---|---|---|---|---|---|---|---|
| | al. (2020), United States | case study of cultural adaptation processes | community adaptation projects; relevant youth age ranges approximately 9–14 years | De-Dah and Thiwáhe Gluwáš’akapi/Strengthening Families Program 10–14 | engaged adaptation conducted over approximately 2 years; adapted versions ranged from 7 to 14 sessions | synthesized in the current report | substances |
| [33] | Murta et al. (2021), Brazil | Pre-experimental mixed-methods study | Quantitative analysis of 126 adolescents and qualitative interviews with 23 adolescents; ages 10–14 years | Brazilian adaptation of the Strengthening Families Program 10–14 | Seven weekly 2-hour meetings: separate parent and adolescent sessions during the first hour and a joint family session during the second hour | Qualitative interviews at 1 week–3 months; quantitative follow-up at 10–12 months | Alcohol, tobacco, cannabis, inhalants, cocaine, and crack |
| [35] | Meyer et al. (2023), Chile | Mixed-methods intervention- | 111 stakeholders: 41 students, 39 parents/caregivers, | Development of the ¡Vamos por Más! parenting program | Planned four 2-hour workshops over two | No effectiveness follow-up; formative development study | Alcohol, tobacco, and other drugs |

| | | | | | | | |
|---|---|---|---|---|---|---|---|
| | | development study | 19 school staff, and 12 experts; grades 5–6, approximately 10–12 years | | academic years, bimonthly virtual engagement, continuing family support, school partnership, and external supervision | | |
| [27] | Haggerty et al. (2023), United States | Secondary outcome analysis of a randomized controlled trial | 220 foster caregiver–youth dyads: intervention 110 and usual-care control 110; youths aged 11–15 years at baseline | Connecting, a self-directed caregiver–youth program | Ten weekly self-directed family modules with video materials; approximately 8 total hours, with brief weekly consultant contact | Postintervention at 3 months and follow-ups at 12 and 24 months; current analysis focused on 24 months | Multiple or unspecified substances and substance-use attitudes |
| [11] | Mehri et | Controlled | 130 parents | Web-based family-centered | Web-based | 2 months postintervention | Cigarettes, alcohol, inhalants, |

| | | | | | | | |
|---|---|---|---|---|---|---|---|
| | al. (2023), Iran | quasi-experimental pretest–posttest study | recruited; 109 analyzed: intervention 61 and control 48; parents of high-school students | empowerment program based on Pender's Health Promotion Model | educational content; exact number and duration of educational modules NR; participants were required to complete at least three educational items | | cannabis, and other substances |
| [28] | Yang et al. (2024), United States | Single-arm mixed-methods feasibility study | Eight justice-involved youth–caregiver dyads; youths aged 14–17 years | Trauma-informed Trust-Based Relational Intervention/Leveraging Safe Adults | Nine 90-minute caregiver sessions, nine 45-minute youth sessions, four 1-hour joint nurture-group sessions, and two 1-hour in-home coaching | Immediate postintervention assessment | Opioids and other substances |

| | | | | | | | |
|---|---|---|---|---|---|---|---|
| | | | | | sessions; additional monthly boosters when release was delayed | | |
| [19] | Berkel et al. (2024), United States | Secondary longitudinal structural equation analysis of an RCT | 667 rural African American families; children aged 11 years at baseline | Strong African American Families (SAAF) | Seven consecutive weekly meetings; concurrent 1-hour caregiver and youth sessions followed by a 1-hour joint family session | Assessments at approximately ages 11, 12, 15, and 16 years | Substance-use-related problems |
| [24] | Skeer et al. (2025), United States | Parallel-group randomized controlled trial with attention | 402 parent–child dyads randomized and 388 analyzed; children in grades | SUPPER: Substance Use Prevention Promoted by Eating Family Meals Regularly | Approximately 20-minute handbook, first intervention | 3, 6, 12, and 18 months | Alcohol, cigarettes, electronic cigarettes/vaping, cannabis, and other drugs |

| | | | | | | | |
|---|---|---|---|---|---|---|---|
| | | control | 5–7, mean age 11.8 years | | session of about 1 hour, second telephone session of about 30 minutes, and two text messages/week for 13 weeks; activities completed over approximately 3 months | | |

Note. NR = not reported; RCT = randomized controlled trial; SAAF = Strong African American Families; SUPPER = Substance Use Prevention Promoted by Eating Family Meals Regularly.

**Supplementary Table S4.** Evidence Mapping of Parental Competency Domains across the Included Publications (N = 32).

| Reference | PC1 | PC2 | PC3 | PC4 | PC5 | PC6 | PC7 | PC8 | PC9 | PC10 | PC11 |
|---|---|---|---|---|---|---|---|---|---|---|---|
| [8] | | T | T | | | | | | T | T | T |
| [9] | | T/M | T/M | T/M | T/M | | | | T/M | | T/M |
| [10] | T | T/M | T/M | | T/M | | T/M | | | T | |
| [11] | T | T | | | | T/M | | T/M | | | |
| [12] | T | T | T | T | T | T/M | T | | | | |
| [13] | | T/M/A[1] | T/M/A[1] | T | | | | | T/M/A[1] | | |
| [14] | | T/M/A[2] | | | T/M/A[2] | | | | T/M/A[2] | | |
| [15] | | T/M | T/M | T/M | | | | | T/M | | |
| [16] | | T/M | T/M | T/M | | | | | T/M | | |
| [17] | | T | T/M | T/M | T/M | | T | | T/M | | |
| [18] | | T | T | T | T | | T | | T | | |
| [19] | | T | T | T | T | | | | T | | T/M/A |
| [20] | | T | | | T | | | | T | | T/M |
| [21] | | T | T | T | T | | | | T | | T |
| [25] | | T/M | T/M | T | T | | | | T/M | | |
| [22] | T | T/M | T/M | T | | T | | T | T | T | |
| [26] | | T/M | T | | | | | | T | | |
| [27] | | T/M | | | T/M | | | | T/M | | |
| [28] | | T/M | | T | | | | | T/M | | |
| [29] | | T | T/M | | T/M | M | | | T/M | | |
| [23] | T/M | T | | | | | | | T/M | | |
| [24] | T | T/M | | T/M | | | | T | T | T | |
| [30] | | T | | | | | | | T/M | | T |
| [31] | | M/A | M/A | M/A | | | | | M/A | | |
| [32] | | M/A | | | | | | | M/A | | |
| [36] | | | M/A | | | | M/A | | M/A | | |
| [33] | | T | T/M | T | | | T | | T/M | | |
| [38] | | | | | | | | | M/A | | |
| [39] | | | | | | | | | M/A | | |
| [37] | | | | | | | T | | T | | T |
| [34] | | T | T | T | T | | | | T | | |
| [35] | | T | T | T | | | T | | T | | |

Note. PC1–PC11 correspond to the parental competency domains defined in Table 2. T = targeted

as intervention content; M = directly measured as a parental competency; A = statistically examined in relation to an adolescent substance-use outcome. Combined codes indicate multiple evidence roles; blank cells indicate that qualifying evidence was not identified. T, M, and A do not indicate statistical significance, causality, or intervention effectiveness.

[1] In Reference [13], A represents a combined family-relationship mediator comprising communication, monitoring, and relational closeness rather than independent associations for each domain.

[2] In Reference [14], A represents a composite positive-parenting construct comprising communication, effective discipline, and mother–child relationship quality rather than independent associations for each domain.
